# Translation and psychometric evaluation of the Swedish versions of the Nuss Questionnaire modified for Adults and the Single Step Questionnaire

**DOI:** 10.1186/s41687-021-00293-2

**Published:** 2021-02-24

**Authors:** Louise Norlander, Jan Karlsson, Agneta Anderzén-Carlsson, Mårten Vidlund, Mats Dreifaldt, Jesper Andreasson, Ann-Sofie Sundqvist

**Affiliations:** 1 Faculty of Medicine and Health, School of Health Sciences, Örebro University, Örebro, Sweden; 2 Department of Cardiothoracic and Vascular Surgery, Örebro University Hospital, Örebro, Sweden; 3 University Health Care Research Center, Faculty of Medicine and Health, Örebro University, Örebro, Sweden; 4 Department of Cardiothoracic Surgery, Skåne University Hospital, Lund University, Lund, Sweden

**Keywords:** Factor analysis, Nuss procedure, Pectus excavatum, Psychometrics, Quality of life

## Abstract

**Background:**

Pectus excavatum (PE) is the most common congenital chest wall deformity. Most individuals with PE suffer from psychosocial problems, with low self-esteem and poor body image. Correctional surgery for PE is available, the most widely used is the Nuss procedure. The Nuss procedure has previously been reported to improve self-esteem, body image and health-related quality of life (HRQoL). To assess HRQoL among individuals with PE, the Nuss Questionnaire modified for Adults (NQ-mA) and Single Step Questionnaire (SSQ) has been developed. The aim of the current study was to translate and culturally adapt NQ-mA and SSQ to fit a Swedish context, and to evaluate the psychometric properties, and validate the culturally adapted versions.

**Methods:**

Individuals who had undergone the Nuss procedure for pectus excavatum were invited to participate in a multicentre study with cross-sectional design. HRQoL was assessed by NQ-mA, SSQ and RAND-36. Psychometric properties for NQ-mA and SSQ were evaluated by content validity index and construct validity (exploratory factor analysis, confirmatory factor analysis, and inter-scale correlations). Known-groups validity, as well as floor and ceiling effects, were evaluated. Internal consistency reliability was examined using Cronbach’s alpha.

**Results:**

In total 236 individuals participated in the study. Content validity index for NQ-mA showed satisfactory results, except for two items. Factor analysis for NQ-mA suggested a two-factor model, with exclusion of two items. Subscales correlated adequately with RAND-36’s domains. Known-groups validity for NQ-mA demonstrated high sensitivity for between-group differences. Ceiling effects were present in several items. Cronbach’s alpha for NQ-mA was .89. Confirmatory factor analysis for SSQ resulted in a three-factor model, with exclusion of five items. However, this model was shown to be unstable through further exploratory factor analysis testing, and no further psychometric tests were conducted for SSQ.

**Conclusion:**

The 10-item Swedish version of NQ-mA was shown to be valid for research and clinical assessment of HRQoL in individuals with pectus excavatum. The Swedish version of SSQ requires revision of items before further validation can be performed.

## Background

Pectus excavatum (PE) is the most common congenital chest wall deformity, with an incidence of 1 in 400 live births, predominately affecting males [1]. PE is characterized by a depression of the sternum and the adjacent costal cartilages. Physical limitations due to the deformity are primarily cardiopulmonary, such as shortness of breath and exercise incapacity, but other symptoms like chest pain may also occur [2]. However, most affected individuals suffer more from psychosocial distress, with low self-esteem and poor body image [3, 4]. Individuals with PE also report decreased health-related quality of life (HRQoL) compared to individuals without PE [5].

Correctional surgery for PE is available. The most widely used is the minimally invasive technique known as the Nuss procedure. This procedure, performed with thoracoscopic guidance, involves placing one or more convex metal bars beneath the sternum, forcing the depression outward [6]. The bars are removed after 3 years when the chest wall has permanently remoulded [7].

It is frequently assumed that correctional surgery for PE is performed solely for cosmetic reasons. Nevertheless, given that individuals with PE may suffer from psychosocial distress and often experience limitations of social activities, surgery can be life changing [8]. In Sweden, the number of correctional surgeries for PE has increased in recent years. Approximately 72 surgeries were performed annually during 2014–2018 and in 2019, 124 surgeries were performed [9]. It is thus essential to evaluate surgical outcomes adequately, not only from the perspective of physical results but also by the effects on HRQoL [10–13]. Previous international studies confirm that the Nuss procedure improves self-esteem and body image, as well as HRQoL [3, 14, 15]. Instruments that measure disease-specific HRQoL among individuals with PE would therefore be helpful for describing how HRQoL is perceived in a Swedish population.

Several questionnaires have been developed to assess HRQoL in individuals with PE. The Pectus Excavatum Evaluation Questionnaire (PEEQ) was developed by Lawson and colleagues [11] for assessment of the impact of the surgery on the HRQoL of children with PE. PEEQ was further modified by Krasopoulos and colleagues [13] into the Nuss Questionnaire modified for Adults (NQ-mA). Krasopoulos also designed the Single Step Questionnaire (SSQ) [13], in addition to NQ-mA, as a simpler one-step instrument to evaluate satisfaction after surgery. NQ-mA and SSQ are the most frequently used instruments for assessment of HRQoL in adults with PE [5, 10, 12, 13, 15–22].

NQ-mA and SSQ are potentially useful instruments to assess HRQoL in adults who have undergone the Nuss procedure in Sweden. However, none of these versions have previously been validated. In order to ensure that HRQoL-instruments provide valid, accurate and interpretable data, it is important to evaluate their psychometric properties [23]. Therefore, the aim of the current study was to translate and culturally adapt NQ-mA and SSQ to fit a Swedish context and to evaluate their psychometric properties and validate the culturally adapted versions.

## Methods

### Data collection and sample

Cross-sectional data were collected from the three Swedish cardiothoracic departments where the largest numbers of Nuss procedures are performed (Skåne University Hospital in Lund, Sahlgrenska University Hospital in Gothenburg and Örebro University Hospital). Data were collected during spring 2019, starting in April. All individuals who had undergone the Nuss procedure between January 2000 and April 2019 were invited to participate in the study. Individuals under the age of 15 at the start of the study were excluded. A total of 420 individuals were included, they were asked to fill out NQ-mA, SSQ, RAND-36 and questions about demographic characteristics.

The questionnaires were sent out by mail and could be answered either by returning the paper questionnaires or via an Internet link. Standard format lay-out was used for the paper questionnaires. For the electronic questionnaires, only minor lay-out modifications were made [24]. The data entry of the paper questionnaires was performed manually, and all registered answers were controlled twice. If a respondent had answered in-between two response options, the item was considered missing.

### Questionnaires

NQ-mA is a 12-item instrument developed to assess the impact of the Nuss procedure on HRQoL [13]. Each item is scored on a four-point Likert scale (Table 1). NQ-mA consists of two domains measuring aspects of psychosocial functioning (PS; items 1–9) and physical functioning (PH; items 10–12) [11]. All items are also summated to a total score, with higher scores indicating better HRQoL.

**Table 1:** Nuss Questionnaire modified for Adults

Question stem	Scoring
1. Looks in general	4. Very happy 3. Mostly happy 2. Mostly unhappy 1. Very unhappy
2. How chest looks without shirt	4. Very happy 3. Mostly happy 2. Mostly unhappy 1. Very unhappy
3. Spending rest of life as chest looks now	4. Very happy 3. Mostly happy 2. Mostly unhappy 1. Very unhappy
4. Other people make fun of you because of your chest looks?	1. Very often 2. Often 3. Sometimes 4. Never
5. Avoid doing things	1. Very often 2. Often 3. Sometimes 4. Never
6. Hides chest	1. Very often 2. Often 3. Sometimes 4. Never
7. Bothered because of the way chest looks??	1. Very often 2. Often 3. Sometimes 4. Never
8. Feels shy/self-conscious because of chest looks???	1. Very often 2. Often 3. Sometimes 4. Never
9. Feels bad about her/himself	1. Very often 2. Often 3. Sometimes 4. Never
10. Has trouble exercising	1. Very often 2. Often 3. Sometimes 4. Never
11. Chest causes shortness of breath	1. Very often 2. Often 3. Sometimes 4. Never
12. Chest is the cause to be tired	1. Very often 2. Often 3. Sometimes 4. Never

The original version of the Nuss Questionnaire modified for Adults, published by: George Krasopoulos, Michael Dusmet, George Ladas, Peter Goldstraw, Nuss procedure improves the quality of life in young male adults with pectus excavatum deformity, *European Journal of Cardio-Thoracic Surgery* 2006; *29*(1): 1–5, doi:10.1016/j.ejcts.2005.09.018. Reprinted by permission of Oxford University Press

SSQ consists of 16 items and was developed for use in addition to NQ-mA at follow-up after surgery. Items 1–7 and 11–15 are scored on a five-point Likert scale, items 8 and 9 are scored on a 10-point visual analogue scale, item 10 is scored on a six-point Likert scale, and item 16 on a three-point scale (Table 2). To calculate the total score, item 8 is subtracted from item 9 and the difference is used as a single score, which is summated with the remaining items [13].

**Table 2:** Single Step Questionnaire

Question stem	Scoring
1. Health in general after the operation	5. Much better now 4. Somewhat better 3. About the same 2. Somewhat worse now 1. Much worse now
2. Exercise capacity after the operation	5. Much better now 4. Somewhat better 3. About the same 2. Somewhat worse now 1. Much worse now
3. Extent that chest looks interfere with pre-operative social activity	5. Extremely 4. Quite a bit 3. Moderately 2. Slightly 1. Not at all
4. Extent that chest looks interfere with post-operative social activity	5. Not at all 4. Slightly 3. Moderately 2. Quite a bit 1. Extremely
5. Satisfaction with the overall post-operative appearance	5. Extremely satisfied 4. Very satisfied 3. Satisfied 2. Dissatisfied 1. Very dissatisfied
6. Bothered by the surgical scars	5. Not at all 4. Very slightly 3. Slightly 2. A little bit 1. A lot
7. Impact operation had to social life	5. Major improvement 4. Improved 3. No change 2. Worse now 1. A lot worse now
8. Pre-operative self-esteem	Score: 1–10
9. Post-operative self-esteem	Score: 1–10
10. Pain during hospital stay	5. None 4. Very mild 3. Mild 2. Moderate 2. Severe 1. Very severe
11. Pain interfering with day-to-day activity now (5 months post-operatively)	5. Not at all 4. Very slightly 3. Slightly 2. A little bit 1. A lot
12. Pain now (5 months post-operative)	5. No 4. Occasionally 3. Mild - no pain-killers 2. Mild - pain-killers 1. A lot
13. Conscious about the metallic bar	5. Not at all 4. Slightly 3. Moderately 2. Quite a bit 1. Extremely
14. Overall satisfaction with the final result	5. Extremely satisfied 4. Very satisfied 3. Satisfied 2. Dissatisfied 1. Very dissatisfied
15. Chest looks different	5. Major improvement 4. Improved 3. No change 2. Worse now 1. A lot worse now
16. Going back, would you have the operation again	10. Yes 5. Un-sure 0. No

The original version of Single Step Questionnaire, published by: George Krasopoulos, Michael Dusmet, George Ladas, Peter Goldstraw, Nuss procedure improves the quality of life in young male adults with pectus excavatum deformity, *European Journal of Cardio-Thoracic Surgery* 2006; *29*(1): 1–5, doi:10.1016/j.ejcts.2005.09.018. Reprinted by permission of Oxford University Press

RAND-36 is a generic HRQoL instrument developed to reflect the World Health Organization’s (WHO) definition of health [25]. RAND-36 consists of 36 items grouped into eight domains: physical functioning (PF), role-physical (RP), bodily pain (BP), general health (GH), vitality (VT), social functioning (SF), role-emotional (RE), and mental health (MH). Domain scores range from 0 to 100, with higher scores indicating better HRQoL.

The participants also filled out a study-specific form eliciting demographic information about sex, age, education, occupation, year of surgery, surgical department, whether bars had been removed, and if so, in what year.

### Translation process

The translation of NQ-mA and SSQ was conducted in four steps, following modified guidelines from the WHO [26]. The translation process included: (1) a forward translation by a certified translator, and (2) a bilingual expert panel that evaluated the adequacy and vocabulary of the translation. A second expert panel with insight into PE, that is, health professionals and individuals living with the diagnosis, assessed the understanding and relevance of the questions and response options. After the two panels had assessed the translation, the number of response options for item 10 in the SSQ was reduced from six to five to fit with the rest of the questionnaire. The responses for item 14 in SSQ were culturally adapted to fit a Swedish context and translated as ‘very satisfied’, ‘satisfied’, ‘neither satisfied nor dissatisfied’, ‘dissatisfied’, and ‘very dissatisfied’. After this, (3) a back translation to English was carried out by two independent certified translators who were unfamiliar with the original wording of the items. The translation process was iterative, and any uncertainties or problems regarding the items were discussed within the research group of health professionals and researchers with experience of instrument development. The back-translated versions of items and response options were sent to the developer, who approved the translations as well as the change in the response options for SSQ item 10.

The fourth step of the translation process was a pilot test. A systematic sample of 42 individuals (10%) from the total study population were included. Participants were asked to fill out the Swedish versions of NQ-mA and SSQ, as well as an evaluation form with questions regarding their comprehension of the questionnaires and whether they found any of the items inappropriate [27]. A total of 24 participants (57%) responded. It took the participants 5–6 min to fill out each questionnaire. The result of the pilot test showed no frequently occurring difficulties with the questionnaires; thus no further changes were made.

### Usability testing

The usability of the electronic questionnaires was evaluated. Eight individuals with different technical skills performed a usability test. In addition, they were asked to evaluate whether the software was compatible with different platforms and operating systems. All reported that the software was easy to use and could be run on a computer, tablet, or mobile phone with different operating systems [24].

### Statistical and psychometric methods

Data were analysed using IBM SPSS Statistics version 25.0 [28] and SAS Software 9.4 [29]. Demographic and clinical characteristics are presented as frequencies and means, along with standard deviations (SD).

#### Content validity

The content validity index (CVI) [30] was calculated to evaluate the adequacy of item content, both for items (I-CVI) and scales (S-CVI) by assessment of relevance. The relevance of items was evaluated by the second expert panel (described above). It consisted of seven people: three of them living with PE, as well as two nurses and two surgeons with experience of individuals undergoing the Nuss procedure. The panel scored relevance on a four-point rating scale with the options: (1) not relevant, (2) somewhat relevant, (3) relevant or (4) very relevant. I-CVI was calculated as the number of experts giving a rating of either 3 or 4 for each item divided by the total number of experts. S-CVI was calculated using the mean of the total I-CVIs for the scale (S-CVI/Ave). An acceptable value for I-CVI is ≥.78, and ≥ .90 for S-CVI/Ave.

#### Construct validity

Exploratory factor analysis (EFA) was conducted to assess the underlying factor structure of the instruments. Confirmatory factor analyses (CFA) were used to evaluate the validity of the extracted factor structure found by EFA.

EFA, using principal axis factoring, was conducted to examine the construct validity of the instruments. As part of the EFA, the Kaiser-Meyer-Olkin test was conducted to measure whether data were suited for factor analysis. Bartlett’s test of sphericity was used to assess whether data were multivariate distributed. Squared multiple correlations were used to compute prior communality estimates. Kaiser criterion (eigenvalue >1), interpretability, and the explained variance (≥50%) were used to determine the number of factors to be retained. Items with a minimum loading of .40 [31] were considered to contribute to a given factor. The promax rotation method was used [32].

CFA, using polychoric correlations and robust maximum likelihood estimation [32, 33], was conducted to test the validity of the factor structure derived by EFA. The following goodness-of-fit indices were used: Root Mean Square Error of Approximation (RMSEA), Standardized Root Mean Square Residual (SRMR), Goodness of Fit Index (GFI), Comparative Fit Index (CFI) and the Tucker-Lewis Index (TLI) [34].

Item-scale convergent validity was examined by corrected item-total correlations, where *r* ≥ .40 indicates acceptable correlations [34]. Spearman’s *rho* was used to assess inter-scale correlations; *r* < .30 was considered a weak correlation, *r* = .30–.49 as moderate, and *r* ≥ .50 as strong [35]. The convergent and discriminant validity were also tested at the scale level against the domains in RAND-36. We hypothesized that the NQ-mA PH subscale would correlate strongly with the PF and RP domains of RAND-36, and that the PS subscale would correlate strongly with VT and SF.

#### Known-groups validity

NQ-mA’s ability to detect differences between groups was examined with known-groups validity, where specific groups were anticipated to score differently. The subgroups were classified depending on how participants responded to item 14 in SSQ: ‘Overall satisfaction with the final result’ (Table 2). Participants who responded ‘very satisfied’ or ‘satisfied’ (subgroup ‘satisfied’) were anticipated to score significantly higher than participants responding ‘neither satisfied nor dissatisfied’, ‘dissatisfied’, or ‘very dissatisfied’ (subgroup ‘dissatisfied’). Cohen’s *d* [35] was calculated to estimate the effect size (ES) of between-group differences. ES was calculated by dividing the mean difference by the pooled standard deviation. ES was considered trivial for *d* < .20, small for *d =* .20–.49, moderate for *d =* .50–.79, and large for *d* ≥ .80. Between-group differences were also tested using the Mann-Whitney U-test, with a statistical significance level of *p* < .05 [34].

#### Floor and ceiling effects

Floor and ceiling effects were calculated as the proportion of participants scoring at the lowest and highest possible levels. Floor or ceiling effects were considered present if ≥50% of the participants answered the lowest or highest possible score respectively for an item [36] or ≥ 15% for scales [37].

#### Reliability

The internal consistency reliability coefficient Cronbach’s alpha (α) was calculated to determine reliability. An α > .70 indicates valid reliability at the group level, whereas α > .90 indicates valid use for individual assessment [34].

## Results

A total of 236 individuals responded to the questionnaires, giving a response rate of 56%. Demographics and clinical characteristics are presented in Table 3.

**Table 3:** Characteristics and demographics

n	236
Sex, n (%)	
Male	194 (82.2)
Female	41 (17.4)
Other	1 (.4)
Age, mean (SD)	25.9 (7.1)
Min-max	15–67
Age at surgery, mean (SD)	19.3 (5.4)
Min-max	10–57
Education, n (%)	
Compulsory	50 (21.2)
Upper secondary	116 (49.2)
Higher vocational education	19 (8.1)
University	50 (21.2)
Missing	1 (.4)
Occupation, n (%)	
Employed	144 (61.0)
Student	80 (33.9)
Job applicant	8 (3.4)
Sick leave	3 (1.3)
Missing	1 (.4)
Department of surgery, n (%)	
Gothenburg	62 (26.3)
Lund	85 (36.0)
Örebro	88 (37.3)
Missing	1 (.4)
Year of surgery, n (%)	
2000–2004	23 (9.7)
2005–2009	40 (16.9)
2010–2014	72 (30.5)
2015–2019	92 (39.0)
Missing	9 (3.8)

### NQ-mA

#### Content validity

I-CVI for NQ-mA showed variable results: item 3 and 12 had a I-CVI of .57 and .28 respectively, while the remaining items had satisfactory results with I-CVI > .86. The S-CVI/Ave for NQ-mA was .82.

#### Construct validity

EFA of NQ-mA suggested a two-factor model. The Kaiser-Meyer-Olkin test was .90 and Bartlett’s test of sphericity showed *p* < .001. Items 1–9, which focus on psychosocial functioning, had loadings ≥ .40 on factor 1, while items 10–12, which measure aspects of physical health had loadings ≥ .40 on factor 2. CFA was conducted to assess the model fit of the two-factor structure. This 12-item model showed a generally poor fit, indicating that the fit between model and data could be substantially improved (Table 4). Inspection of modification indices showed that the covariance between the residuals for items 2 and 3 caused the largest misfit. Very strong correlation between items 2 and 3 (*r* = .84) was also observed, indicating redundancy. Item 2 was seen as a crucial item covering one of the main difficulties of living with PE, and thus contributing unique information about HRQoL in individuals with PE. Item 3 was therefore removed. Subsequently, a two-factor model with 11 items was tested. The model fit improved significantly, but fit indices indicated that further refinement was needed (Table 4). The covariance between the residuals for items 1 and 2 was the largest cause of misfit, and as item 2 was still considered crucial, item 1 was excluded. The two-factor model for the remaining 10 items showed overall acceptable goodness of fit values (Table 4). A chi-square difference test showed a significant improvement in model fit for the 10-item model. T-values for the factor loadings were significant (*p* < .001) and the standardized factor coefficients ranged between .49 and .92 (Table 5). Since two items had been removed, EFA was performed to test the factor structure of the 10-item, two-factor model. A Kaiser-Meyer-Olkin value of .90 and Bartlett’s test of sphericity of *p* < .001 showed that it met the requirements. Eigenvalues, explained variance, and factor loadings had satisfactory values for the 10-item version (Table 5).

**Table 4:** Confirmatory factor analysis; Goodness-of-fit indices for Nuss Questionnaire modified for Adults and Single Step Questionnaire

Fit indices	NQ-mA, 2 factors	NQ-mA, 2 factors	NQ-mA, 2 factors	SSQ, 1 factor	SSQ, 3 factors	SSQ, 3 factors
Number of items	12	11	10	14	12	10
Items excluded	–	3	1, 3	13	6, 10, 13	3, 6, 10, 12, 13
Chi-square/df	4.59	3.2	2.25	7.14	5.38	3.4
RMSEA (90% CI)	.13 (.11–.15)	.10 (.08–.12)	.08 (.05–.10)	.17 (.15–.18)	.14 (.12–.16)	.10 (.08–.12)
SRMR	.05	.05	.04	.11	.09	.06
GFI	.83	.90	.93	.72	.84	.91
CFI	.90	.94	.97	.66	.84	.93
TLI	.87	.92	.96	.60	.79	.91

Reference values for acceptable fit: Chi-square/df < 3, RMSEA < .10, SRMR < .08 and GFI, CFI, TLI > .90

*CFA* confirmatory factor analysis, *df* degrees of freedom, *RMSEA* root mean square error of approximation, *CI* confidence interval, *SRMR* standardized root mean square error, *GFI* goodness of fit index, *CFI* comparative fit index, *TLI* Tucker Lewis index

**Table 5:** Nuss Questionnaire modified for Adults: construct validity, missing, responses, floor/ceiling effects, and reliability

Factor	1	2					
Eigenvalue	5.26	1.55	Missing (%)	Floor/Ceiling^a^ (%)	Corrected item-total correlation^b^	Cronbach’s α^c^	α if item deleted
Cumulative variance %	49.42	60.88					
	Loadings EFA (CFA)						
PS			1.8	6.5/51.5		.91	
2	.72 (.77)	.07	2.5	10.2/24.6	.71		.90
4	.44 (.49)	.11	2.1	.4/85.6	.47		.92
5	.92 (.88)	−.02	2.1	5.1/60.2	.85		.89
6	.91(.90)	−.09	1.7	8.9/53.4	.86		.88
7	.89 (.90)	−.00	1.7	7.2/45.8	.86		.88
8	.94 (.92)	−.05	1.3	7.2/54.7	.87		.88
9	.52 (.59)	.12	1.3	5.9/34.7	.55		.92
PH			2.1	4.7/51.0		.77	
10	.05	.76 (.80)	1.7	4.2/55.9	.58		.71
11	−.01	.74 (.72)	1.3	6.8/30.1	.60		.69
12	.03	.70 (.71)	3.4	3.0/64.8	.63		.66

*PS* psychosocial subscale, *PH* physical subscale, *EFA* exploratory factor analysis, *CFA* confirmatory factor analysis

Items with loadings > .40 were considered to contribute to a factor. ^a^ Number of subjects scoring at the lowest or highest possible scale level. ^b^ *r* > .40 was acceptable. ^c^ α > .70 indicates valid use for group assessment, α > .90 indicates valid use for individual assessment

Corrected item-total correlations for the 10-item version resulted in overall acceptable values (Table 5). The psychosocial (PS) and the physical (PH) subscales correlated strongly with the total score of the 10-item NQ-mA (Table 6). All inter-scale correlations between the NQ-mA and RAND-36 scales are presented in Table 6. PH correlated strongly with PF and RP of RAND-36, as hypothesized, as well as with GH. PS had moderate correlations with VT and SF. Moderate correlations were also seen between PS and GH, RE and MH.

**Table 6:** Inter-scale correlations between Nuss Questionnaire modified for Adults and RAND-36

	NQ-mA		RAND-36							
Scale	PS	PH	PF	RP	BP	GH	VT	SF	RE	MH
NQ-mA										
PS	1.00	.41	.20	.14	.17	.37	.43^a^	.39^a^	.32	.45
PH	.41	1.00	.**56**^**b**^	.**50**^**b**^	.45	.**55**	.42	.40	.28	.33
Total	**.93**	.**69**	.40	.32	.31	.**50**	.**50**	.46	.35	.48

Spearman’s *rho* for correlations; *r* < .30 is considered weak correlation, *r* = .30–.49 moderate, *r* ≥ .50 strong. Strong correlations are bolded. Correlations with the same lowercase letter were hypothesised to correlate.

*PS* psychosocial, *PH* physical, *PF* physical functioning, *RP* role-physical, *BP* bodily pain, *GH* general health, *VT* vitality, *SF* social functioning, *RE* role-emotional, *MH* mental health

#### Known-groups validity

Known-groups analysis showed significant differences between the two subgroups for all scales (Table 7). Cohen’s *d* showed large ES for PS and the total score for the 10-item NQ-mA, indicating good sensitivity to detect differences between groups. ES for PH was small (ES = .44), demonstrating less sensitivity to between-group differences.

**Table 7:** Nuss Questionnaire modified for Adults: known-groups analysis

Scale	Satisfied^a^, *n* = 167 Mean (SD)	Dissatisfied^a^, *n* = 65 Mean (SD)	*p*-value^b^	ES^c^
PS	24.86 (3.28)	18.48 (4.86)	< .001*	1.54
PH	10.16 (1.85)	9.22 (2.35)	.004*	.44
Total	35.02 (4.40)	27.71 (5.99)	< .001*	1.39

Higher score indicates better health status. ^a^Subgroups classified based on response to item 14, SSQ. ^b^Mann-Whitney U-test for independent group differences. *Significant at level < .05. ^c^ES, Effect Size according to Cohen’s *d*, *d* < .20 = trivial ES, .20–.49 = small, .50–.79 = moderate, ≥ .80 = large

#### Floor and ceiling effects

Floor and ceiling effects are presented in Table 5. Six items (4–6, 8, 10 and 12) had high ceiling effects (> 50%), and also for the PS and PH scales (> 15%).

#### Reliability

Cronbach’s alpha for PS indicated valid use for individual assessment, while PF is valid on the group level (Table 5). Cronbach’s alpha for the total scale was .89, representing nearly acceptable value for individual assessment.

### SSQ

#### Content validity

CVI for SSQ was satisfactory with I-CVI > .86 for all items and a S-CVI/Ave of .97.

#### Construct validity

As item 8 is subtracted from item 9 to calculate the total score, the difference was used as a single item for the psychometric tests. Item 13 was excluded as 61.4% were missing, since these participants had already removed their bar(s). CFA for SSQ was conducted with a one-factor model of all the remaining 14 items. However, this model generally showed poor fit (Table 4) and EFA was therefore conducted to assess what factor structure was supported by the data.

EFA, performed on the 14-item SSQ, resulted in a four-factor model with acceptable values for the Kaiser-Meyer-Olkin test of .83, and Bartlett’s test of sphericity of *p* < .001. However, item 10 did not load on any factor and was excluded. For the remaining 13 items, EFA suggested a three-factor model. However, item 6 had a weak loading of .31 and so was excluded. EFA of the remaining 12 items again indicated a three-factor model (Table 8). Factor 1 consisted of five items, all focussed on appearance. Factor 2 consisted of four items reflecting aspects of physical health, and factor 3 consisted of three items measuring psychosocial aspects and self-esteem.

**Table 8:** Single Step Questionnaire: exploratory factor analysis

Factor	1	2	3
Eigenvalue	4.69	1.18	1.04
Explained variance, cumulative (%)	39.07	48.87	57.5
Item	Loadings		
5	**.99**	−.09	−.14
14	**.93**	−.02	−.07
15	**.82**	−.16	.16
4	**.61**	.15	−.36
16	**.51**	.12	.13
12	−.11	**.81**	−.10
11	−.02	**.79**	−.22
1	.09	**.52**	.30
2	.07	**.51**	.24
3	−.31	−.15	**.86**
8–9	.27	−.07	**.64**
7	.29	.19	**.50**

SSQ item 6, 10 and 13 excluded after exploratory factor analysis exploration

Loadings ≥ .40 are bolded and were considered to contribute to a factor

CFA of the 12-item three-factor model showed improved goodness-of-fit indices, although the indices were still not acceptable. As modification indices indicated problems with item 3 and 12, these items were excluded, resulting in a 10-item model with fairly acceptable fit. However, further EFA testing of the ten remaining items showed that the three-factor structure was unstable. In addition, the loss of information because of the excluded items was extensive, and it was questionable whether the reduced SSQ was still able to provide reliable information about HRQoL. As a result, no further psychometric testing of SSQ was carried out.

## Discussion

The present study aimed to translate and evaluate the psychometric properties of the Swedish versions of NQ-mA and SSQ. As individuals with PE are reportedly suffering from psychosocial and physical limitations due to their chest wall deformity, it is important to adequately measure HRQoL to assess the impact of correctional surgery. The present study found support that a 10-item Swedish version of NQ-mA could be used to assess HRQoL in individuals who have undergone the Nuss procedure. However, the Swedish version of SSQ showed weak construct validity due to the inability to find a stable factor solution.

The results of the psychometric analyses conducted on the Swedish version of NQ-mA showed the 10-item version to be valid for assessment. However, the NQ-mA showed questionable content validity based on CVI results. Lynn [38] suggests removing all items with fair I-CVI or lower ratings in order to achieve content-valid items before distributing the instrument to participants. However, we used a different strategy, and the decision to retain or delete items was instead based on the aggregated results of the psychometric analyses. Thus, items 3 and 12 with fair and low I-CVI, respectively, were retained until psychometric analyses had been evaluated.

NQ-mA item 12 had the highest rate of missing responses, which may imply that participants had difficulty responding to this item. Whether the chest wall issue causes fatigue depends on the extent to which the individual experiences physical limitations due to the deformity. Factor analysis confirmed that item 12 loaded on the PH factor, and as this item was considered to contribute unique information on how PE affects physical HRQoL, it was retained. Another reason for retaining item 12 is that the PH domain consists of only three items, which is the minimum recommended number of items per factor [39].

EFA suggested a two-factor structure of NQ-mA, which is consistent with the results of factor analysis of the Turkish version [40]. CFA could not, however, confirm the model without allowing covariance errors to correlate. In the present study, two items (1 and 3) were excluded, a decision based on both statistical and theoretical grounds [31]. Item 1 correlated strongly with item 2, indicating that these items measure the same aspect of the latent variable and that one item is sufficient. Item 2 was considered important because it measures one of the most frequently reported issues among individuals with PE [14, 41] and thus contributes unique information about HRQoL. Item 1 focuses on overall appearance, not solely regarding the chest wall, which makes the item too general for assessment of disease-specific HRQoL [42]. Item 3 had questionable I-CVI ratings. It may be difficult for participants to respond to a question regarding the future. Furthermore, the correlated error covariances between item 2 and both items 1 and 3 indicate that some other aspect beyond what is measured by the PS domain is influencing the association between these variables [32]. This is an additional reason to exclude items 1 and 3. The suggested 10-item version of NQ-mA is hereafter referred to as NQ-mA-10.

The correlation between the PS and PH subscales of NQ-mA-10 was moderate, suggesting that the scales measure different domains (Table 6). As hypothesized, strong correlations were seen between the PH subscale and RAND-36’s domains PF and RP, providing evidence for convergent validity [34]. PH correlated strongly with the domain GH as well, suggesting that PH measures aspects of general health. Small to moderate associations were seen for PH and the remaining domains, implying discriminant validity. PS had weak correlations with three of the physical health scales (PF, RP and BP), supporting discriminant validity. However, only moderate correlations were seen between PS and the VT and SF domains of RAND-36, which had been hypothesized to correlate strongly. PS also showed moderate associations with GH, RE and MH. This result suggests that the PS scale measures aspects of HRQoL that are not fully consistent with the RAND-36 domains [43]. Thus, moderate correlations between PS and the mental health scales of RAND-36 (VT, SF, RE and MH) indicate that PS measures unique aspects of psychosocial health in individuals with PE that are not captured by a generic HRQoL instrument. Hence, generic and disease-specific instrument should be used in conjunction, as they provide complementary information on HRQoL.

Ceiling effects were present in several items of NQ-mA-10, and both subscales had ceiling effects well above the acceptable cut-off value. This result can be explained by the cross-sectional design of the study and the fact that HRQoL was measured only postoperatively. In many cases the questionnaire was filled out several years after surgery. The ceiling effects can thus simply demonstrate that participants experience only minor problems because of the positive effects of the surgery. However, a longitudinal study with assessment both before and after surgery is required to accurately evaluate the ceiling effects. Responsiveness can also be evaluated with such a design, which was not possible in the present study.

The Swedish version of SSQ had satisfactory content validity. However, several problems were noted during the factor analysis process, especially concerning item content. Two of the excluded items (3 and 10) ask participants to recall experiences from the past, prior to surgery. Items constructed in this way may be influenced by recall bias as health status memory is often unreliable, especially after long recall periods [34]. In this regard, item 8 is troublesome as well, as it refers to self-esteem prior to surgery. According to Fayers and Machin [34], questions regarding current health status are preferable. Since SSQ is designed to measure HRQoL after surgery, it may be more beneficial to merge item 8 and 9 into one item asking about the difference in self-esteem experienced after surgery.

The wording of SSQ item 12 was considered sub-optimal. This item refers to general sensations of pain that are not solely associated with the chest wall. Consequently, the wording is too unspecific to assess disease-specific HRQoL in individuals with PE. It has not been established whether the same issues regarding item 12 have arisen in the assessment of other SSQ versions. Casamassima and colleagues [12] used a modified version of SSQ to evaluate satisfaction with surgical results after bar removal. Five items (7, 8, 9, 13 and 15) were excluded, but this modified version was never validated. SSQ has been translated into several other languages [10, 17, 18, 20], but without psychometric evaluations. Thus any issues regarding SSQ items in other versions remain unknown.

Despite improved and almost satisfactory goodness-of-fit indices for the 10-item factor model of SSQ, further EFA testing showed that the factor structure was unstable. Additionally, the loss of information due to the excluded items was considered to be excessive. In conclusion, the wording and content of items need extensive revision before further validation can be carried out.

The present study did not assess test-retest reliability, which is a limitation. It is important to evaluate whether SSQ and NQ-mA scores are stable over time and can provide reproducible results. As the original SSQ was validated through test-retest [13], a comparison to assess whether the Swedish version was consistent with the original version would have been possible.

The Swedish version of SSQ could not be validated due to inadequate construct validity. Further reconstruction of items and a new validation study is required before the Swedish SSQ may be available. Additionally, assessment of HRQoL in individuals with PE prior to surgery is needed to further evaluate the Swedish versions of both NQ-mA and SSQ.

## Conclusion

The results of the present study show that the Swedish NQ-mA-10 has adequate reliability, sensitivity and validity, indicating that the psychometric properties of this translated and culturally adapted version are sound. The instrument is thus valid for assessment of HRQoL among individuals who have undergone the Nuss procedure for PE in Sweden.

## Data Availability

The datasets generated and analysed during the current study are not publicly available to ensure the privacy of the participants, but are available from the corresponding author on reasonable request.

## Abbreviations

BP: Bodily pain
CFA: Confirmatory factor analysis
CFI: Comparative fit index
CVI: Content validity index
EFA: Exploratory factor analysis
ES: Effect size
GFI: Goodness of fit index
GH: General health
HRQoL: Health-related quality of life
I-CVI: Content validity index for item
MH: Mental health
NQ-mA: Nuss Questionnaire modified for Adults
PE: Pectus excavatum
PEEQ: The Pectus Excavatum Evaluation Questionnaire
PF: Physical functioning
PH: Physical
PS: Psychosocial
RE: Role emotional
RMSEA: Root mean square error of approximation
RP: Role physical
S-CVI: Content validity index for scale
SD: Standard deviation
SF: Social functioning
SRMR: Standardized root mean square
SSQ: Single Step Questionnaire
TLI: Tucker-Lewis index
VT: Vitality
WHO: World Health Organization
